# Dynamics-driven allosteric stimulation of diguanylate cyclase activity in a red light-regulated phytochrome

**DOI:** 10.1016/j.jbc.2024.107217

**Published:** 2024-03-24

**Authors:** Quang Hieu Tran, Oliver Maximilian Eder, Andreas Winkler

**Affiliations:** 1 Institute of Biochemistry, Graz University of Technology, Graz, Austria; 2 BioTechMed Graz, Graz, Austria

**Keywords:** photoreceptor, photocycle, signal integration, HDX-MS, c-di-GMP

## Abstract

Sensor-effector proteins integrate information from different stimuli and transform this into cellular responses. Some sensory domains, like red-light responsive bacteriophytochromes, show remarkable modularity regulating a variety of effectors. One effector domain is the GGDEF diguanylate cyclase catalyzing the formation of the bacterial second messenger cyclic-dimeric-guanosine monophosphate. While critical signal integration elements have been described for different phytochromes, a generalized understanding of signal processing and communication over large distances, roughly 100 Å in phytochrome diguanylate cyclases, is missing. Here we show that dynamics-driven allostery is key to understanding signal integration on a molecular level. We generated protein variants stabilized in their far-red-absorbing Pfr state and demonstrated by analysis of conformational dynamics using hydrogen-deuterium exchange coupled to mass spectrometry that single amino acid replacements are accompanied by altered dynamics of functional elements throughout the protein. We show that the conformational dynamics correlate with the enzymatic activity of these variants, explaining also the increased activity of a non-photochromic variant. In addition, we demonstrate the functional importance of mixed Pfr/intermediate state dimers using a fast-reverting variant that still enables wild-type-like fold-changes of enzymatic stimulation by red light. This supports the functional role of single protomer activation in phytochromes, a property that might correlate with the non-canonical mixed Pfr/intermediate-state spectra observed for many phytochrome systems. We anticipate our results to stimulate research in the direction of dynamics-driven allosteric regulation of different bacteriophytochrome-based sensor-effectors. This will eventually impact design strategies for the creation of novel sensor-effector systems for enriching the optogenetic toolbox.

There are numerous molecular mechanisms in nature that integrate environmental signals to respond to nutrient availability, temperature changes, and light conditions. At the protein level, signal integration relies on the communication between sensory domains or allosteric regions and effector modules or functional sites, respectively, either through their non-covalent interaction or through the inclusion of both elements in one polypeptide chain. Common modes of action at the molecular level include the binding of diverse ligand molecules (*e.g.*, G protein-coupled receptors (1)) or pH variation-induced changes in protonation states (2) affecting amino acid side chain conformations (3) and/or protein dynamics (dynamics-driven allostery) to allosterically regulate the target functionality. Alternatively, also covalent modifications of amino acids, for example, phosphorylation (4), or bound cofactors (5), as well as changes in redox chemistry (6) can be employed to alter protein structure and/or conformational dynamics.

Historically, allosteric signaling pathways were frequently described as a continuous set of residues linking allosteric and active sites (7), however, more recently the concept of dynamics-driven allostery (8) put population shifts of conformational substates at the center stage of allosteric signaling (9). An intuitive model of dynamics-driven allostery has been described for protein kinases in analogy to the functioning of violins (10), and a functional correlation has been observed in a variety of systems and even implicated in protein evolution (11). Interesting systems where even structural analogies to violins hold true (12) are the family of red light-sensing bacteriophytochromes (13, 14).

In the context of advancing our understanding of molecular details involved in allosteric signal integration, photoreceptors, like the above-mentioned phytochromes, offer advantages in their functional characterization as the light stimulus that switches them from either OFF to ON position or vice versa can be precisely controlled in time and space. Therefore, correlating the properties of the corresponding transitions between dark- and light-adapted states of the sensory domain with the conformational dynamics of the whole sensor-effector couple as well as with the functional properties of the output modules offers great potential to learn more about the molecular details involved in light signal integration and processing. Especially since red-light responsive systems have also attracted attention in the field of optogenetics, where red light might solve the penetration problem associated with other wavelengths (15), an improved understanding of the molecular properties will be beneficial for developing interesting new sensor-effector combinations.

Despite the accumulated knowledge of functional elements involved in signal integration in phytochromes (13, 14, 16, 17, 18), the coupling of early sensory signal integration steps around the bilin chromophore with the distant effector domains are only poorly understood and hence generalization between different sensor-effector combinations is rarely successful. Typically, signaling is initiated by photon absorption of the red-absorbing phytochrome state—so-called Pr. In bacteriophytochromes, biliverdin (BV) bound to the central GAF domain of the tripartite PAS-GAF-PHY photosensory module isomerizes upon red light absorption and transitions from its 15*Z* to its 15*E* configuration resulting in a 180° flip of the BV D-ring. *Via* a route of typically short-lived intermediates (17, 18), the far-red absorbing Pfr state is populated with moderate quantum efficiencies. Figure 1*A* shows an example of such a canonical phytochrome with Pr (dark) and Pfr (red illuminated) spectra (19). Along this Pr to Pfr transition, residues lining the flipped D-ring alter their side-chain conformations (13), the chromophore rotates in its binding pocket (20) and secondary structure elements change their orientation (N-terminal segment (NTS) (21, 22, 23)) or even change their structure from beta-hairpin to alpha-helix (24, 25, 26, 27). While this could be considered as part of a classical allosteric pathway description, recent observations hint at a more complex dynamic interplay of multiple elements of the photosensory module affecting properties of the photocycle, and also the functionality of the associated effector (28).

**Figure 1:**
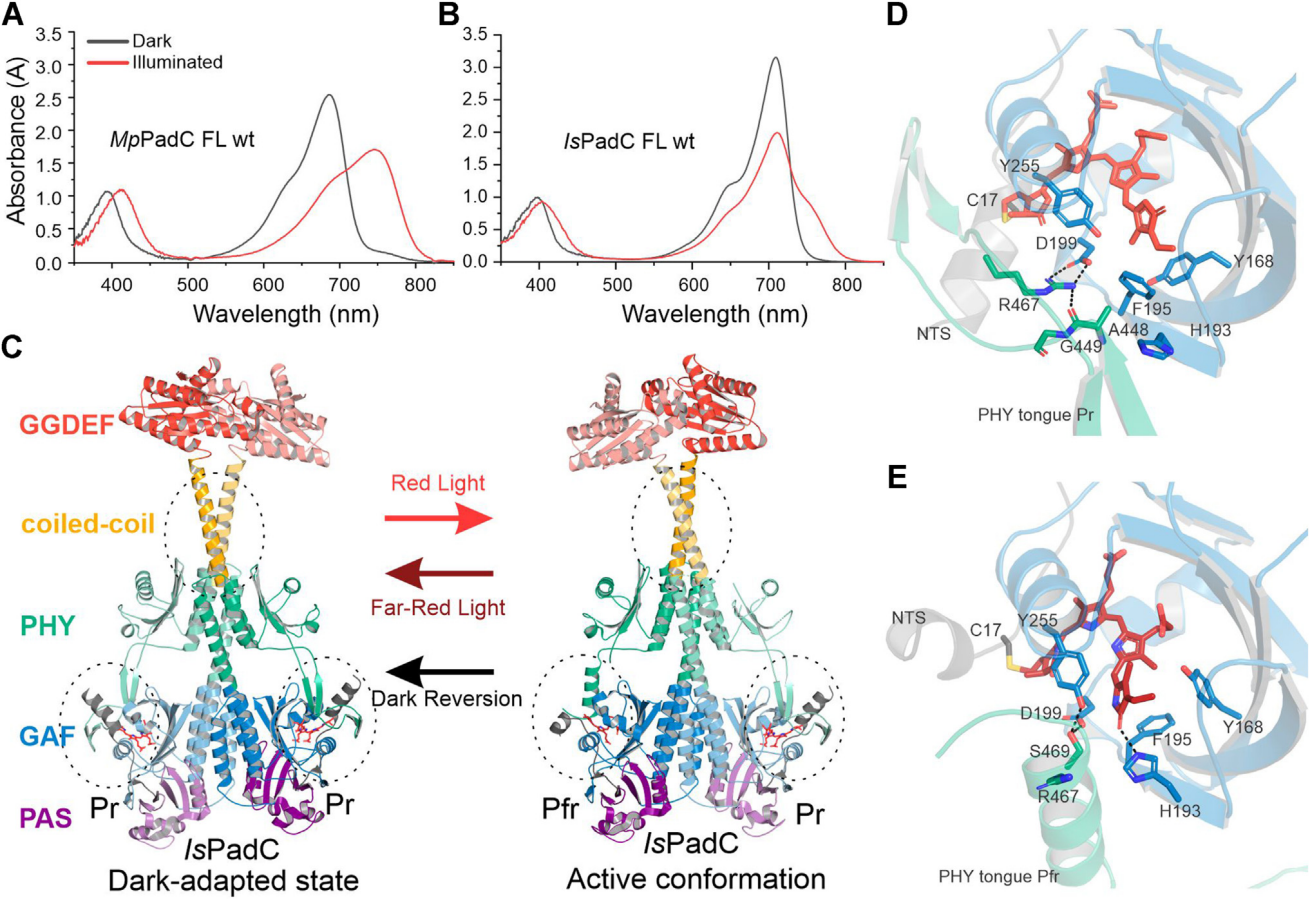
**Spectral and structural properties of PadCs.** UV/Vis absorption spectra of (*A*) *Mp*PadC featuring canonical Pr and Pfr states in dark and illuminated states, respectively, and (*B*) *Is*PadC with its non-canonical spectral behavior upon illumination. *Black lines* represent dark-adapted states and *red lines* represent light-adapted states. *C*, full-length structures of *Is*PadC in its dark-adapted state (pdb: 5LLW) (*left*) and an active conformation of a protein variant (pdb: 6ET7) (*right*). Structural elements affected during activation are highlighted with *dashed ellipses* and supposedly correlate with structural rearrangements upon light activation (*arrows*). Individual domains are colored according to *dark gray* for the N-terminal segment (NTS), *blue* for the GAF domain, *green* for the PHY-domain, *yellow* for the coiled-coil, and *red* for the GGDEF domain. *D*, close up of the *Is*PadC Pr architecture corresponding to chain A of pdb 5LLY. *E*, close up of the *Is*PadC Pfr architecture corresponding to chain A of pdb 6ET7. For better visibility, the cartoon representations are shown with partial transparency. Both panels feature selected critical residues of the Pr to Pfr transition, residues targeted by mutagenesis in this study, and the biliverdin cofactor in *stick* representation. Hydrogen bonding interactions are indicated with *black dashes*. *Is*PadC, *Idiomarina species* A28L phytochrome-activated diguanylate cyclase.

The latter observations have been described for the well-characterized bacteriophytochrome system, *Idiomarina species* A28L phytochrome-activated diguanylate cyclase (*Is*PadC), that allows red light control of diguanylate cyclase (DGC) activity *via* the coupled GGDEF domain (12). *Is*PadC is an interesting model system from the field of bacteriophytochromes as it features non-canonical light state spectra (*i.e.*, only partial Pfr population upon red illumination, Fig. 1*B*) that might be linked to the functional importance of structural asymmetry in the regulation of DGC activity (Fig. 1*C*) (24). Previously, chimeras of *Is*PadC and its homolog *Mp*PadC, with the latter featuring a canonical light state spectrum (Fig. 1*A*), demonstrated that canonical *versus* non-canonical spectra are not defined by specific residues or contacts, but rather by an interplay of the PHY-tongue, the NTS and most likely a so far uncharacterized element in the PAS-GAF bidomain (28).

In order to further address molecular details of the non-canonical spectral properties of *Is*PadC we set out to stabilize the Pfr conformation by introducing amino acid substitutions affecting photocycle properties. On the one hand, we chose Pfr-stabilizing variants described in other phytochrome systems and, on the other hand, we addressed highly conserved, functionally important residues as controls to benchmark how *Is*PadC compares to other bacteriophytochrome model systems in this respect.

We identified two variants that stabilize the Pfr state of *Is*PadC by more than three orders of magnitude. Nevertheless, the proteins feature a non-canonical light state spectrum, supporting previous conclusions that the rapid reformation of Pr is not responsible for the spectral properties after illumination (21). In addition, we generated one protein variant exhibiting almost instantaneous thermal recovery to Pr, thereby preventing the accumulation of Pfr. Strong stimulation of enzymatic activity is still observed in this variant, supporting the hypothesis that a structural/functional heterodimer with a single protomer of *Is*PadC in the Pfr conformation is sufficient for the stimulation of DGC activity (24).

By combining spectroscopic data with functional assays of the effector domain in the context of full-length bacteriophytochromes and complementing these analyses with structural information obtained from hydrogen-deuterium exchange coupled with mass spectrometry (HDX-MS) data, we provide comprehensive insights into how individual amino acid changes can result in pronounced alterations of protein structure and conformational dynamics of phytochromes that extend over the whole sensor-effector assembly. Thereby, our results further support the involvement of dynamics-driven allostery in signal integration of phytochromes, a critical insight for considering new designs of sensor-effector combinations in the field of optogenetics.

## Results

### Expression and purification of *Is*PadC variants

In order to stabilize the Pfr conformation in *Is*PadC, we generated amino acid substitutions in highly conserved regions of the PHY-tongue, such as residue Gly449 in the conserved phytochrome W(A/G)G motif and residue Arg467 in the PRXSF motif. In addition, we investigated critical residues in the BV binding environment by replacing His193, which changes its conformation between Pr and Pfr states (21), or Asp199 of the strictly conserved DIP motif. All of these residues have been shown to strongly impact the photocycle properties of phytochromes in diverse backgrounds (20, 21, 26, 29, 30, 31, 32). A close-up of the interactions of these residues in the proximity of the BV cofactor is shown in Figure 1, *D* and *E*.

Gly449 is located in the conserved W(A/G)G motif that forms a characteristic 90° turn in the Pr hairpin structure of the PHY tongue (33). Importantly, the backbone carbonyl of the Ala448-Gly449 peptide bond hydrogen bonds to the guanidinium group of Arg467, stabilizing the Pr-defining salt bridge character between the respective arginine and DIP Asp199 (Fig. 1*D*). Replacing Gly449 with glutamate (G449E) disrupts this characteristic structure by destabilizing the beta-hairpin character of the tongue through steric clashes, that do not influence the alpha-helical tongue Pfr conformation (Fig. 1*E*). The above-mentioned interaction partner, Arg467, belongs to the highly conserved PRXSF motif that in Pr interacts with Asp199 of the DIP motif and constitutes the beginning of the characteristic Pfr helix upon illumination (Fig. 1*E*). Therefore, replacing Arg with Ser (R467S) prevents the anchor-like docking of the Pr conformation to the biliverdin pocket in Pr and should also favor Pfr-like conformations.

His193 is structurally close to the above-mentioned interactions and was shown to change its rotamer conformation during the Pr to Pfr transition forming a hydrogen bond with the D-ring carbonyl in Pfr. We replaced this amino acid with other phytochrome-specific amino acids at the corresponding position—Leu and Gln (H193L and H193Q). The highly conserved Asp199 of the DIP motif is positioned at the heart of the photochemical events and connects to the PHY-tongue *via* the Arg467 salt bridge in Pr and probably stabilizes the positive charge on ring D in Pfr (34, 35). It was replaced by either Leu or Asn (D199L and D199N) to test the influence of charge and hydrogen bonding character.

All variants expressed in comparable amounts to the wild-type (wt) protein and were efficiently purified *via* Ni-NTA chromatography, reverse Ni-NTA chromatography after His tag removal, and subsequent size-exclusion chromatography. The elution profiles during gel filtration revealed that all variants are dimeric as observed for the wild-type protein. During initial purifications, it was noted that D199L showed variable amounts of apoprotein content (50–70%) and to a lesser extent this was also observed for R467S (20% apo) and D199N (5% apo). Therefore, we supplemented free BV after lysis during all purifications and tested full loading with BV by intact mass measurements of purified samples. This confirmed that all proteins were fully loaded and covalently bound to biliverdin.

### Spectral characterization

**Figure 2:**
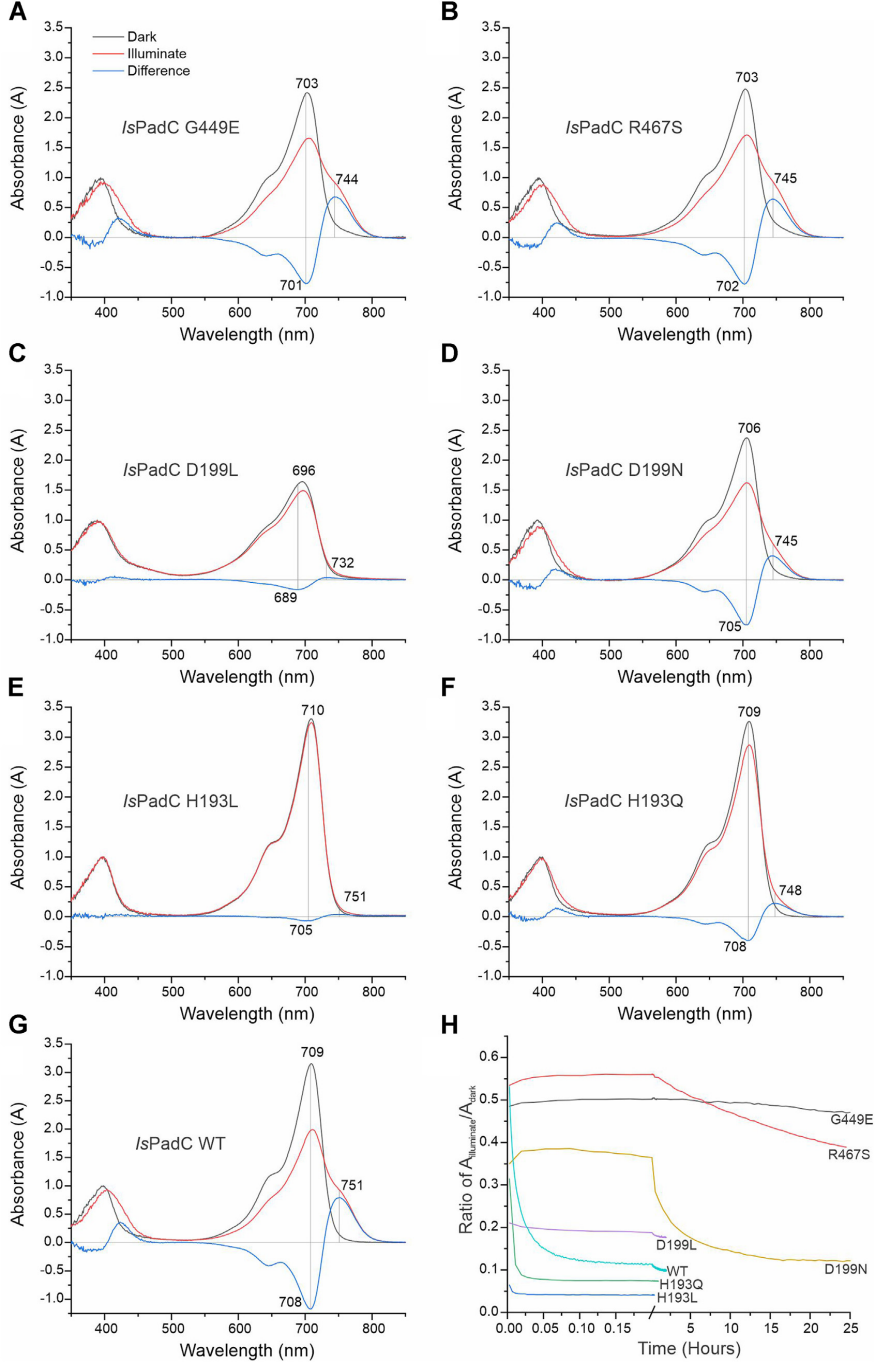
**Spectral characterization of *Is*PadC variants.***A*–*G*, UV/Vis absorption spectra. As shown in panel (*A*), but representative for all panels (*A*–*G*), the *black line* represents dark-adapted states, the *red line* represents light-adapted states, and the difference spectrum (*light minus dark*) is in *blue*. The *gray lines* indicate the maxima and minima of the difference spectra. Spectra are scaled based on the maximum Soret-band spectrum of each variant's dark state spectrum. *H*, dark recovery of *Is*PadC WT and variants. The absorbance ratio at the maximum and minimum wavelengths of the difference spectra was plotted *versus* recovery time. *Is*PadC, *Idiomarina species* A28L phytochrome-activated diguanylate cyclase.

All characterized variants featured spectral signatures of the Pr state after purification in non-actinic light conditions. However, a closer inspection revealed interesting differences in their respective absorption maxima and extinction coefficients of the Soret-relative to the Q-band. Considering no drastic changes in the BV environment, *i.e.*, all variants featuring the characteristic ZZZssa configuration in Pr, the Soret band extinction coefficient is typically less affected by the environment than the Q-band. Therefore, Figure 2 shows all spectra normalized to the Soret absorption maximum. This revealed that substitutions at position His193 (Fig. 2, *E* and *F*) retain the ∼3-fold higher Q-band absorption of the WT, whereas a reduction to ∼2.5-fold is observed for G449E, R467S and D199N (Fig. 2, *A*, *B*, and *D*). D199L shows an even more pronounced reduction in its Soret extinction coefficient (Fig. 2*C*). Hand-in-hand with the reduced extinction coefficient, the absorption maxima of Pr shift towards the blue; WT-like 710 and 709 nm for H193L and H193Q, respectively, 706 nm for D199N, and 703 nm for both R467S and G449E. The strongest shift was observed for D199L with a Pr absorption maximum at 696 nm.

Interesting additional distinctions emerged when we addressed the effect of red-light illumination. Similar to the light-state spectrum of WT *Is*PadC, the light-adapted states of the G449E and R467S variants displayed non-canonical mixed populations of Pfr and Pfr-like contributions (21) with only minor blue-shifts compared to the WT. Importantly, however, the illuminated states are very stable and recover to only 5% or 30% over 24 h for G449E and R467S, respectively. Interestingly, G449E forms stable higher oligomeric states upon illumination that can be observed by size exclusion chromatography (Fig. S1), likely due to similar non-native interface interactions as observed for the *Is*PadC PAS-GAF construct (21). R467S behaves similar to G449E in many respects—as detailed below—however, does not show indications of such oligomeric species.

Full-length *Is*PadC constructs H193L and H193Q, on the other hand, behaved oppositely, and only minor indications of Pfr contributions were detected. Due to their fast thermal recoveries (Fig. 2*H*), the majority of the Pfr signal is lost within the dead time of data acquisition. Even under continuous illumination, only minor indications of a Pfr population can be observed (Fig. S2). Since this substantial acceleration of thermal recoveries was somewhat unexpected upon substitution of His193 with residues occurring in related phytochromes, we also addressed their effect in the context of the PAS-GAF core construct of *Is*PadC (Fig. S3). Interestingly, in this setting, both variants also feature substantially accelerated thermal recoveries, however starting from an almost non-reverting PAS-GAF wild-type (21). Since the effects of the amino acid replacements are also not identical to the full-length context, this suggests that the observations do not reflect an isolated exchange of amino acid chemistry, but rather a network of interactions with surrounding structural elements.

The third group of variants focused on Asp199 of the phytochrome DIP motif. While D199N retained some functionality and showed indications of a non-canonical light state spectrum, it recovered only slowly to its respective dark state. In contrast, D199L featured a completely impaired photocycle and red-light illumination results in partial bleaching of the Soret absorption band. A similar trend was observed for the fluorescent properties of these variants. Since substitutions of the DIP Asp have been shown to increase fluorescence in other model systems (31, 36), we also addressed the fluorescent properties of the corresponding *Is*PadC variants (Fig. S4). D199L showed a roughly 10-fold increase in fluorescence intensity, whereas D199N, with a 4-fold increase and interesting subtle differences in excitation and emission spectra, is again in between WT and D199L properties.

Interestingly, three out of the six variants characterized showed unexpected spectral behavior during dark state recoveries. Immediately after switching off the red-light illumination, initial measurements showed a subtle increase in the Pfr/Pr ratio (Fig. 2*H*), potentially indicating slow progression through photocycle intermediates after initial chromophore excitation. This was observed for the slow recovering variants R467S, G449E, and D199N and was confirmed with different measurement regimes not to be an artifact of the measuring light intensities.

### Enzymatic characterization

To address the influence of amino acid substitutions in the biliverdin environment on long-range signal integration in the effector domain of *Is*PadC, we measured the formation of c-di-GMP in dark and light-adapted states of selected variants. In this context, we focused on the strongest Pfr-stabilizing variants (G449E and R467S) as well as H193L, which showed the least spectral Pfr contribution. In addition, we included D199L as benchmarking residue for a non-photoactive variant.

**Figure 3:**
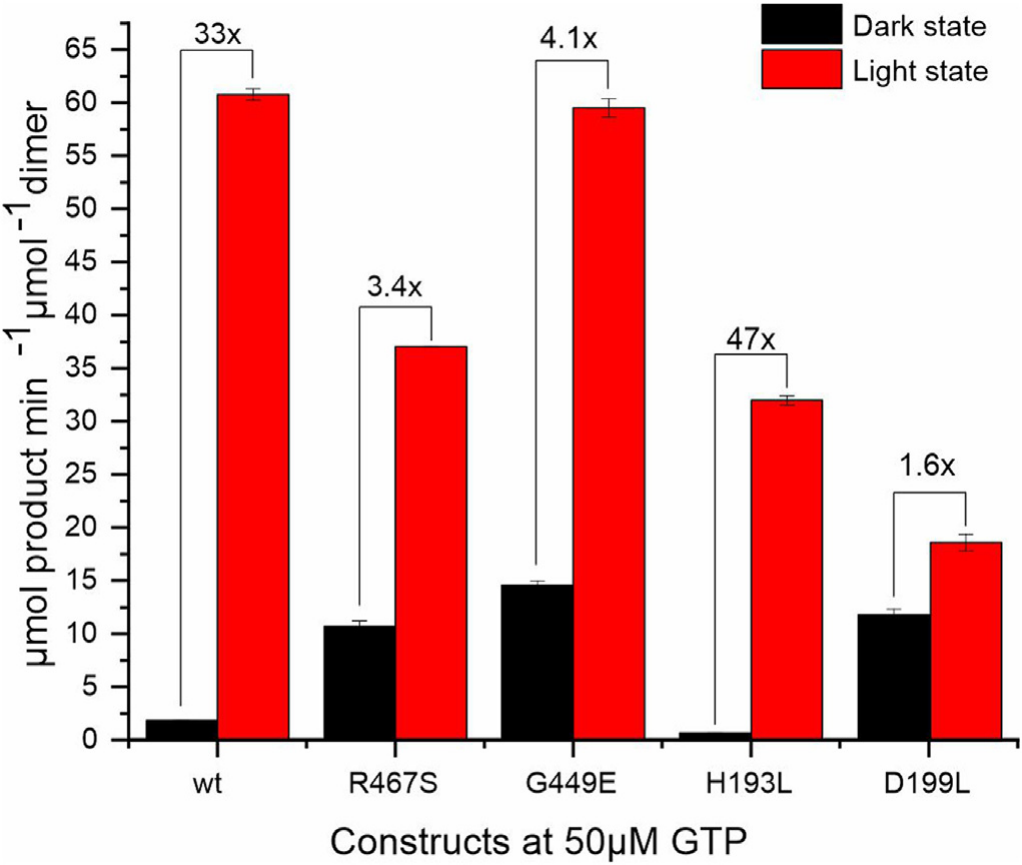
**The specific activity of substrate turnover per dimer of i*****diomarina species*****A28L phytochrome-activated diguanylate cyclase and variants.** Shown are values obtained for a substrate concentration of 50 μM under dark and light-adapted conditions. Substrate dependencies of all tested constructs are shown in Fig. S5. The approximate fold changes are indicated as *brackets* above the bars. Error bars show the error of the estimate for linear fits of c-di-GMP production weighted by the standard deviation from three experimental replicates.

Comparing the GTP conversion of these variants to WT revealed quite unexpected insights (Fig. 3). D199L, for example, which features a bleached Pr-like absorption and no longer photoactivates, showed a roughly 5-fold increased dark state activity and only negligible activity stimulation upon illumination. Interestingly, the Pfr stabilizing variants G449E and R467S, with almost normal Pr spectra in the dark, also featured increased dark state activities. In contrast to D199L, however, these variants showed increased enzymatic activity in their light states with moderate fold-changes of roughly 3.5 and 4.5 for R467S and G449E, respectively. H193L, on the other hand, showed an almost wild-type-like fold-change of ∼30-fold, with slightly lower absolute dark and light state activities compared to the WT, albeit featuring only very low steady-state levels of Pfr. Since no significant drop in enzymatic activities was observed in all tested cases, the variants appear to be dimeric as required for the condensation of two GTP molecules at the active site between two GGDEF domains even at the concentration of 0.1 μM in the enzymatic assay.

While Figure 3 compares only the variants' product formation at 50 μM substrate, the substrate concentration with the highest GTP conversion for *Is*PadC WT, the conversion of substrate to c-di-GMP of the variants G449E, R467S, and D199L displays indications of substrate inhibition similar to the WT (Fig. S5). Interestingly, only variant H193L did not exhibit substrate inhibition under the conditions tested.

Since there is no obvious connection between photocycle properties and substrate inhibition, because of its WT-like dynamic range, and anyway only small expected changes in deuterium uptake due to its negligible Pfr population, we omitted H193L from the subsequent analyses of conformational dynamics and their involvement in defining dark state activities and fold-changes upon illumination.

### HDX-MS reveals dynamics in solution

To probe the conformational dynamics of *Is*PadC variants we used hydrogen-deuterium exchange coupled with mass spectrometry (HDX-MS). Analyzing amide proton exchange dynamics provides insights into the secondary structure stability of functional elements throughout the whole protein and thereby provides potentially valuable information with respect to signal integration between sensor and effector domains (37). Among the tested variants, R467S and G449E are amino acid substitutions in the PHY-tongue, which changes conformation during the Pr to Pfr transition. While the two variants disrupt different molecular properties of Pr tongue stabilization, they result in similar spectral profiles, substantially slowed-down dark recoveries, and comparable enzymatic activity profiles with residual light activation. In contrast, Asp199 of the PASDIP motif is a critical amino acid for the structure and function of phytochromes and its substitution with Leu has drastic consequences, as shown by the spectral characteristics, increased dark-state activity and rather low fold-changes of enzymatic activity in the D199L variant.

**Figure 4:**
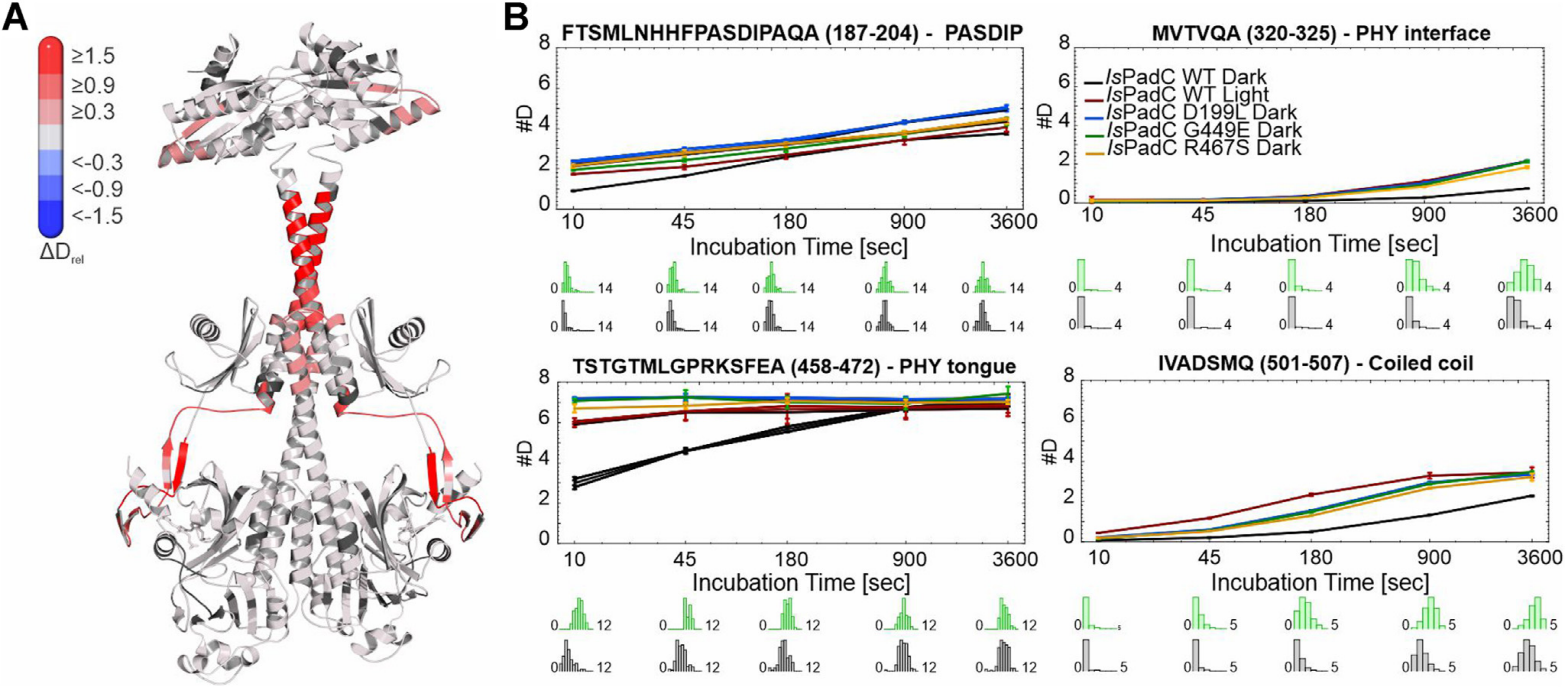
**HDX-MS results.***A*, differences in deuterium uptake for the *Is*PadC G449E minus *Is*PadC WT comparison after 180 s of deuterium incorporation mapped onto the structure of *Is*PadC. All analyzed time points are shown side-by-side in Fig. S7 and this panel is reused therein. *B*, deuterium uptake plots of selected peptides of the PASDIP motif, the PHY dimer interface, the PHY-tongue, and the coiled-coil sensor-effector linker. Shown are the relative amounts of deuterium incorporation plotted against exchange time. Individual lines correspond to the relative deuterium levels of the wild type in dark- and light-adapted states (*black* and *red*, respectively) and dark-adapted states of variants D199L (*blue*), G449E (*green*) and R467S (*orange*). Error bars indicate the sample standard deviation of deuterium incorporation from experimental triplicates as analyzed by Hexicon 2 (59). The distributions below the uptake curves correspond to the software estimated number of deuterium atoms incorporated at each exchange time point. *Is*PadC, *Idiomarina species* A28L phytochrome-activated diguanylate cyclase.

Full details of HDX-MS measurements can be found in Table S3 (HDX-MS data) and Figs. S6–S9. Focusing on the most important structural elements in the main text, Figure 4, shows a representative difference in deuterium incorporation of one exchange time point mapped onto the structure of *Is*PadC WT (Fig. 4*A*) as well as representative deuterium uptake plots of peptides in important regions with an overlay of incorporation kinetics of the analyzed proteins. Most pronouncedly, PHY-tongue destabilization in *Is*PadC can be seen in all functional states of the tested variants as well as upon illumination of WT *Is*PadC (Figs. 4*B*, S6, S7, and S8). Similarly, we observed an increase in deuterium incorporation in the PASDIP motif (187–204), PHY interface (320–325), and the coiled-coil sensor-effector linker (501–507) for all of the variants compared to the wild type in the dark, which resembles the behavior of the wild type protein upon illumination (Figs. 4 and S9*D*). This increase in deuterium incorporation correlates with the increase in enzymatic activity for all the variants in the dark. While directly linking conformational dynamics of individual regions of the sensory module with enzymatic activity likely is an oversimplification, the results indicate that changes in conformational dynamics of functionally important regions of the sensory domains go hand-in-hand with functional property changes of the effector.

In addition to the comparison of dark state conformations of all variants, we also addressed the effect of red-light treatment on the variants. Upon illumination, both G449E and R467S feature additionally elevated deuterium exchange, particularly noticeable in the PHY interface (300–350) and the coiled-coil region (amino acid 500–530), as compared to their respective dark-adapted states (Fig. S9, *B* and *C*). In the context of light activation, these observations are in line with the signal integration pathway involving increased conformational dynamics at the dimer interface required for coiled-coil transitions between different registers (12). In contrast, D199L showed a subtle reduction in deuterium exchange across the protein, except for the DIP region (Fig. S9*A*). This suggests that D199L is impaired in long-range signal integration and that local effects of BV activation cannot be integrated by the tongue, the PHY interface, and the coiled-coil. Eventually, this correlates with the limited fold-change of 1.6-fold in enzymatic activity.

## Discussion

While the diguanylate cyclase phytochrome from *Idiomomarina* (*Is*PadC) has provided interesting insights toward long-range signal integration mechanisms of bacteriophytochromes (12, 21, 23, 24, 28), it has so far not been addressed by extensive mutagenesis studies to assess its model system character in comparison to other prototypic members in the field. Especially the peculiar non-canonical illuminated state spectra are of concern when considering *Is*PadC as a model system. It should be noted, however, that actually a substantial fraction of analyzed bacteriophytochromes feature such mixed Pfr spectra (19, 32, 38, 39, 40, 41) and that clearly not in all cases fast thermal recoveries to Pr can be the sole explanation for this behavior.

In this study, we targeted three functional elements, previously demonstrated to be important for phytochrome function and especially photocycle properties, with the intention to test the model system character of *Is*PadC. First of all, the nearly invariant aspartate residue (Asp199 in *Is*PadC) of the DIP motif (13) was replaced by either leucine, to obtain an almost isosteric side chain lacking the charge and hydrogen bonding character, or asparagine, to only remove the negatively charged character. Nearly identical to *Deinococcus radiodurans* phytochrome (*Dr*BphP), the D199L variant completely lost its capability to form Pfr, and red-light illumination instead rather induced a subtle bleaching of the Pr Q-band (31). Similar observations were made for other systems, like *Agrobacterium fabrum* phytochrome (Agp1, (42)), the bathyphytochrome from *Pseudomonas aeruginosa* bacteriophytochrome (*Pa*BphP being locked in Pr upon D194A substitution, (43)), cyanobacterial members of the phytochrome family (Cph1, (30)) and even plant representatives like *Arabidopsis thaliana* phytochrome B (*At*PhyB, (44)). Generally, these results are interpreted as proteins locked in their respective meta-Rc intermediate states, where a full transition to Pfr is blocked by the missing charge of the conserved aspartate (31, 42). Also, the properties of D199N in *Is*PadC can be nicely correlated with observations in *Dr*BphP (31), where retaining the hydrogen bonding capability results in partial Pfr formation. In addition, reduced Pr Q-band extinction coefficients were observed for DIP-aspartate substitutions in *Dr*BphP, likely reflecting subtle changes in the bilin binding pocket organization. Along the same line, the increased fluorescent properties of the two DIP-variants mimic observations in *Dr*BphP (31) and the broad excitation spectra could also reflect the heterogeneity of bilin conformations in the pocket of the Pr-like state. Interestingly, initial purification of the corresponding variants in *Is*PadC revealed only partial loading with biliverdin especially for D199L, which is not observed for the wild-type protein. While supplying excess biliverdin during the purification allowed full reconstitution of holoprotein, the observation that the correct organization of the bilin binding pocket is hampered, especially by the central D199L substitution, suggests that the BV environment is also important for efficient cofactor loading. Similar observations were made previously also for *Dr*BphP (31, 45), where protoporphyrin IX binding competes with BV addition in the respective D207A variants.

The second region of interest focused on a residue strongly affecting photocycle properties in several phytochromes *via* mediating tongue and cofactor interactions. In *Is*PadC the corresponding amino acid, His193, was shown to change rotamer conformation upon transitioning from Pr to Pfr (21, 24) and probably stabilizes Pfr by coordinating the flipped biliverdin D-ring similar to observations in *Dr*BphP (25, 46). Also in other model systems, like *Xanthomonas campestris* bacteriophytochrome (*Xcc*BphP) the equivalent Leu193 residue is critical for maintaining the characteristic Pfr-stabilized bathyphytochrome character (47) and replacing Leu with Gln or Asn generates Pr-favoring variants. Interestingly, even the opposite chemical change introduced by the Q188L variant in another bathyphytochrome, *Pa*BphP, resulted in a similar Pr stabilizing effect and only very slow thermal reversion to its Pfr-ground state (20). These apparent contradictions can only be explained by a more extensive network of interactions and the importance of conformational dynamics in the binding pocket defining the relative stabilities of Pr and Pfr. For *Is*PadC we generated the H193N and H193L variants and observed that both amino acid substitutions greatly enhanced thermal recovery to Pr. The H193L variant is so fast that only during constant illumination clear Pfr characteristics can be observed (Fig. S2). While this result might initially not seem too important, one advantage of the *Is*PadC system, however, is its straightforward functional readout based on the DGC activity of the GGDEF effector domain. Importantly, DGC activity was substantially increased upon illumination of the H193L variant even though also under constant illumination only less than 10% Pfr can be formed. In the context of long-range signal integration, this observation supports mixed Pfr/intermediate-state and apparently also Pfr/Pr “hetero”-dimers as functional units in red light activation of PadCs (24). Since recently Pr/Pfr heterodimers were also structurally observed for *Stigmatella aurantiaca* phytochrome 1 (*Sa*BphP1, (48)) this feature could be more generally relevant in the field of bacteriophytochromes than initially expected. In the context of plant phytochromes, phyA Pr/Pfr heterodimers are probably responsible for the very low fluence response (49) and also phyB Pr/Pfr dimers feature distinct biophysical properties with functional implications for plant morphogenic development (50).

The third functionally important element of phytochromes addressed by amino acid substitutions is the PHY-tongue. Based on observations of substantially increased Pfr stabilities, we chose the corresponding amino acid substitutions in *Is*PadC: G449E (*cf.*, *Dr*BphP G453E (51) and *Xcc*BphP G454E (26)) and R467S (*cf.*, *At*PhyB R582A (29)). Indeed, Pfr stabilities were greatly enhanced for both *Is*PadC variants and thermal recoveries slowed down by three orders of magnitude (Fig. 2*H*). However, the non-canonical spectral properties of *Is*PadC were retained in both the G449E and the R467S variant (Fig. 2, *A*, *B*, and *G*). Therefore, Pfr stability is clearly not the reason for incomplete Pfr formation in *Is*PadC, but rather defined by so far unknown properties of the bilin environment. Based on chimeras between *Is*PadC and a close homolog with canonical Pfr spectra it can be concluded that neither the PAS-GAF core nor the NTS or the PHY-tongue themselves define this property, but rather the dynamic interplay of the latter two in concert with a so far unidentified region in the PAS-GAF region is responsible for defining Pr and Pfr stabilities (28). One candidate for this region might be the loop region preceding the DIP motif. Since the effect of identical amino acid substitutions of His193 shows different outcomes in the PAS-GAF and full-length *Is*PadC context, this region could be involved in tuning overall conformational dynamics of this region close to the NTS, the PHY-tongue and the BV-binding pocket.

In principle, HDX-MS also enables the identification of different conformations within an ensemble measurement provided that the corresponding exchange kinetics differ substantially. This was actually observed upon illumination for peptides of the PHY-tongue, the coiled-coil and the central helical spine in the case of an *Is*PadC variant stabilizing the activated Pr/Pfr heterodimer structure (24). The characteristic broad or bimodal isotope distributions, however, were not observed in the analyses of the protein variants studied here; most likely due to the generally increased conformational dynamics observed in these regions already in the corresponding dark states.

Interestingly, even though both Pfr-stabilized variants feature typical Pr spectra, their dark state enzymatic activities are substantially increased (Fig. 3). Considering the above-mentioned role of dynamics in defining Pr and Pfr stabilities, it might not seem surprising that changes in conformational dynamics can be coupled to downstream signaling elements. Considering the concept of dynamics-driven allostery (10) the allosteric communication between the sensor and effector should actually be affected by the perturbation of dynamics close to BV chromophore. In fact, even the non-photochromic D199L variant shows an increase in enzymatic activity in the dark (Fig. 3). These potentially unexpected observations are also interesting for other systems, especially plant phytochromes, where many interesting amino acid substitutions are based on mutational screening. Apparently, even non-photochromic variants can show increased physiological output activities as observed for example for “Pfr phenotypes” in otherwise photoinsensitive constructs of *At*PhyB Y276H (52), a substitution that also in bacteriophytochromes does not allow photochromicity (Y176H in *Dr*BphP (31) and Y176H in Cph1 (53)).

To further address the coupling of sensor and effector domains and the importance of dynamics-driven allostery we employed HDX-MS. The results obtained for the comparison of three variants with elevated dark state enzymatic activities revealed a clear correlation of increased conformational dynamics in the proximity of the cofactor binding site, the PHY-tongue, the PHY dimer interface, and the coiled-coil sensor effector linker region. All of these effects are also observed upon light activation of *Is*PadC and hence demonstrate how changes in conformational dynamics throughout the protein are employed to tune the enzymatic activity of the effector domain. Supporting this hypothesis, the additional increase in conformational dynamics of the coiled-coil linker observed for the Pfr-stabilized variants, G449E and R467S, supports the residual fold-changes observed upon illumination of the corresponding variants (Fig. 3). In contrast, D199L does not feature this increase in dynamics upon illumination, which fits to the observation of only minor stimulation of enzymatic activity (Fig. 3) and the fact that no indications of Pfr are present (Fig. 2*C*). In essence, these results support previous mechanistic hypotheses that increased PHY-tongue dynamics increase the conformational flexibility of the PHY dimer interface and its directly connected coiled-coil linker (12). This results in the population of different coiled-coil registers, one of which stimulates DGC activity as recently also seen for other GGDEF domains (54). In the overall context of phytochromes, the appreciation of dynamics-driven allostery complements insights into effector activation obtained by other approaches, like NMR (55), crystallography (24), or cryo-EM (48, 56).

In summary, *Is*PadC serves as an interesting additional bacteriophytochrome model system that features non-canonical light states. It will be interesting to follow up on which regions define the conformational dynamics responsible for these partial Pfr spectra and whether they play a functional role in defining structural asymmetry in the activated bacteriophytochrome dimer. As far as signal integration is concerned, dynamics-driven allostery is a key concept to better understand molecular mechanisms of phytochrome function and HDX-MS serves as a powerful technique to address the effect of illumination or other physiologically relevant perturbations. Combining these insights with other biophysical tools will help to improve our understanding of phytochrome functioning and harnessing the full potential of red light-regulated systems in the field of optogenetics.

## Experimental procedures

### Protein expression and purification

Plasmid pET-M11 *Is*PadC containing the coding sequence of the PadC homolog from *Idiomarina* sp. A28L (WP_007419415), as described initially in (12), was used as the template for PCR-based mutagenesis to generate the *Is*PadC variants H193L, H193Q, D199L, D199N, R467S, and G449E based on the protocol of Liu and Naismith (57). The primers used can be found in Table S1.

The protein variants were produced in *Escherichia coli* BL21 (DE3) containing the pT7 ho1 helper plasmid coding for the heme-oxygenase (HO-1) from *Synechocystis* sp. PCC6803 and a chloramphenicol resistance marker (12). Protein expression and purification of the (His)_6_-tagged holoproteins complexed with biliverdin IXα were performed under non-actinic, dim green light conditions. At an OD_600_ of 0.4, cultures were supplemented with 10 mg l^−1^ δ-aminolevulinic acid, and incubation shifted from 37 °C to 16 °C. At an OD_600_ of 0.8, 0.25 mM isopropyl-β-D-thiogalactopyranoside (IPTG) was added to induce protein expression, and cultures were incubated for an additional 16 h.

Cells were harvested by centrifugation at 5000 RCF at 4 °C. For protein purification, cells were resuspended in a lysis buffer (Table S2) containing lysozyme (100 μg ml^−1^) and lysed by sonication (2 × 10 min, 50 W, Labsonic LU, 0.7 s duty cycle, ice water cooling). Lysates were clarified by centrifugation at 39,000 RCF at 4 °C for 1 h. To achieve consistent holo-protein formation, we added excess biliverdin-IXα to a final concentration of 30 μM and incubated on ice for 1 h with gentle stirring. Holoproteins were then purified by gravity flow affinity chromatography on a Ni^2+^-Sepharose matrix. After 10 column volumes of wash buffer (Table S2) containing 30 mM imidazole, the holoproteins were finally eluted by elution buffer (Table S2) containing 250 mM imidazole. (His)_6_-tags were cleaved by TEV protease in a weight ratio of ∼1:16 for TEV protease/substrate in dialysis buffer (Table S2) during overnight dialysis at 4 °C. The TEV protease and the cleaved (His)_6_-tags were separated from the proteins by a reverse Ni^2+^-Sepharose step, and the proteins of interest were collected in the flow-through. After concentration using centrifugal filters (Amicon MW 30000 cut-off, 4000 RCF), the holoproteins were further purified by size-exclusion chromatography using a Superdex 200 increase 10/300 Gl column equilibrated in gel-filtration buffer (Table S2). The purified dimer fractions were concentrated, flash-frozen in liquid nitrogen, and stored at −80 °C for further characterization.

### Ultraviolet-visible absorption spectroscopy

Ultraviolet-visible absorption spectra were recorded on a Specord 200plus spectrophotometer with a 10 ms integration time and a scan rate of 100 nm s^−1^. The protein samples were diluted to 2 μM in gel filtration buffer (Table S2) and equilibrated at 20 °C in the dark. To fully populate the dark-adapted Pr conformation, a 5-min exposure to far-red light (780 nm, Thorlabs) followed by a prolonged (at least 1 h) incubation in darkness was performed before taking measurements. To obtain light-state absorption spectra, samples were illuminated under red light (660 nm, 20.9 μW mm^−2^, Thorlabs) for 5 min before measurement. Placing the cuvette and starting data acquisition results in a delay of 5 to 10 s for the actual data measured.

Pr-state recovery was monitored at the minimum and maximum Q-band absorption of the difference spectrum (light *minus* Pr). The measurements were conducted using a Specord 200plus spectrophotometer with a 10 ms integration time and different time intervals adjusted to the recovery kinetics observed during initial tests in order to minimize the effect of the measuring light on recovery kinetics.

For variants showing a Pfr-destabilization, light state absorption spectra were additionally recorded under constant red-light illumination (660 nm, 20.9 μW mm^−2^, Thorlabs) using a CCD-based Specord S300 Vis spectrophotometer. The measuring light intensity of the spectrophotometer was minimized using a neutral density filter (ND 1.0) placed between the light source and the sample cuvette.

### Fluorescence measurements

Measurements were conducted on an RF-5301PC spectrofluorimeter (Shimadzu) using a 150 W Xenon lamp as a light source. Sample concentrations were adjusted to 2 μM in gel filtration buffer (Table S2). Diluted samples were equilibrated at room temperature and measured in triplicates with 780 nm illumination in between to enrich Pr state populations. General settings of the fluorimeter were slit widths of 5 nm for the excitation and the emission channels, scanning speed “super” (50 nm s^−1^), and sensitivity “high”. For emission spectra, 685 nm was chosen as excitation wavelength, whereas excitation spectra were recorded at 740 nm emission. Rescaling of excitation spectra was performed by multiplying by two for clearer visualization. Finally, all spectra were normalized to 100% signal intensity for the D199L emission spectrum.

### Enzyme kinetics

To characterize GTP conversion to c-di-GMP, we adapted the high-performance liquid chromatography (HPLC) protocol described previously (19). Briefly, purified protein samples at a concentration of 0.2 μM were equilibrated at 20 °C under low-intensity, non-actinic light conditions (indirect green light). Protein variants featuring a stabilization of the photo-activated state were initially treated by a 5-min exposure to far-red light (780 nm, Thorlabs) followed by a prolonged 1-h incubation in darkness. This was done to fully populate the dark-adapted state of the samples.

GTP turnover was initiated by mixing 35 μl protein solution at 0.2 μM with 35 μl GTP at various concentrations (100 μM, 400 μM, and 1 mM) in reaction buffer (Table S2) and incubated at 20 °C. The reactions of the dark-adapted state of each variant were prepared in a low-intensity, non-actinic green light. In contrast, the light-adapted state reactions required continuous red-light illumination (660 nm, 20.9 μW mm^−2^, Thorlabs) following a 1-min pre-illumination. Each reaction was performed in triplicate and stopped at specific time points by quenching in 50 mM EDTA pH 8.0, followed by immediate thermal inactivation at 95 °C for 1 min. Time points for analysis of enzyme kinetics were selected only in the linear range of initial velocities with less than 10% GTP turnover. The substrate and product were separated from the denatured protein by centrifugation at 27,000 RCF for 10 min. The nucleotides were separated on a reversed-phase HPLC column (Prontosil 120-3-C18 ACE-EPS 4.6 mm by 100 mm, Bischoff) using an aqueous mobile phase (10 mM K_2_HPO_4_ pH 7.0, 1 mM EDTA, 6% MeOH) under isocratic conditions at 20 °C. None of the characterized variants in this study shows the formation of the intermediate pppGpG.

Analysis of relative peak areas of c-di-GMP *versus* GTP was used to quantify initial velocities of GTP turnover. The amount of c-di-GMP formed during reactions of dark and light-adapted states was plotted against the corresponding time points. The sample standard deviation (SD) of reaction point triplicates contributed to the error estimation of the linear fit used to calculate the initial rate of product formation. The standard error (SE) of the linear regression estimate is shown as an error indicator. All kinetic data are normalized to the concentration of the dimeric proteins.

### HDX-MS

Aliquots of 3 μl at 100 μM protein concentration were used for deuterium labeling reactions. To this end, protein samples were equilibrated at 20 °C either in dim-green light or with parallel red-light illumination for 2 min (660 nm, 20.9 μW mm^−2^, Thorlabs) and then diluted 20-fold in D_2_O buffer containing 10 mM HEPES (pD 7.0/pH_read_ 6.6), 150 mM NaCl, and 10 mM MgCl_2_. Aliquots of 6 μl were removed at various time points (10 s, 45 s, 3 min, 15 min, and 60 min) and quenched by equivalent volumes of 200 mM ice-cold ammonium formic acid (pH 2.5) and then flash-frozen in liquid nitrogen. For HPLC-MS analysis, frozen samples were quickly thawed with 50 μl 200 mM ice-cold ammonium formic acid (pH 2.5) and then injected into a cooled HPLC system as described previously (58).

Briefly, protease digestion was carried out on an immobilized pepsin column (BEH Enzymate, Waters) at 10 °C with a flow rate of 0.3 ml min^−1^. Subsequently, peptide fragments were desalted using a C18 trap column (Shim-pack GISS-HP(G), Shimadzu) and were separated during a 4.25 min acetonitrile gradient (from 10% to 45%) containing 0.6% (v/v) formic acid on a reversed-phase column (Shim-pack Arata Peptide, Shimadzu). Species eluting from the column were infused into an Impact II ESI-Q-TOF (Bruker) mass spectrometer. The analysis and quantification of the deuterium incorporation were performed with the Hexicon2 software package (59). The carry-over level was less than 10%. The back exchange was estimated based on exchange characteristics of short peptides with high redundancy and apparent complete deuteration. Accounting for the fast back-exchanging first two amide positions as well as the lack of exchangeable amide protons for proline residues allowed the estimation of ∼30% back exchange.

### Intact mass measurements

2 μl of protein preparations at 10 μM were desalted on a Shim-pack Scepter C4-300 (G) column (3 μM) by washing with 1% ACN in the presence of 0.1% formic acid. Subsequently, increasing concentrations of acetonitrile (1–95%) eluted the proteins into an Impact II ESI-Q-TOF (Bruker) mass spectrometer. Protein signatures in the mass spectra were integrated and deconvoluted using the maximum entropy function of DataAnalysis (Bruker).

## Data availability

All data needed to evaluate the conclusions of the paper are present in the paper and/or the Supporting information file. All data used in the analyses is available in the public repository of Graz University of Technology under doi: 10.3217/86ez3-3q642.

## Supporting information

This article contains supporting information.

## Conflict of interest

The authors declare that they have no conflicts of interests with the contents of this article.
