# Supplementary material for: Dynamics-driven allosteric stimulation of diguanylate cyclase activity in a red light-regulated phytochrome
Source: J Biol Chem. 2024 Mar 24;300(5):107217. doi: 10.1016/j.jbc.2024.107217 (PMC11035067; doi:10.1016/j.jbc.2024.107217)
Supplement: Supporting Figures S1–S9 and Tables S1 and S2 [file mmc1.pdf]

Dynamics-driven allosteric stimulation of diguanylate cyclase activity in a red-light-regulated phytochrome

Quang-Hieu Tran[1], Oliver Maximilian Eder[1], and Andreas Winkler[1,2]

Affiliations

[1] Graz University of Technology, Graz, Austria

[2] BioTechMed Graz, Graz, Austria

Supporting information

Running title

Mutational analysis of a phytochrome diguanylate cyclase

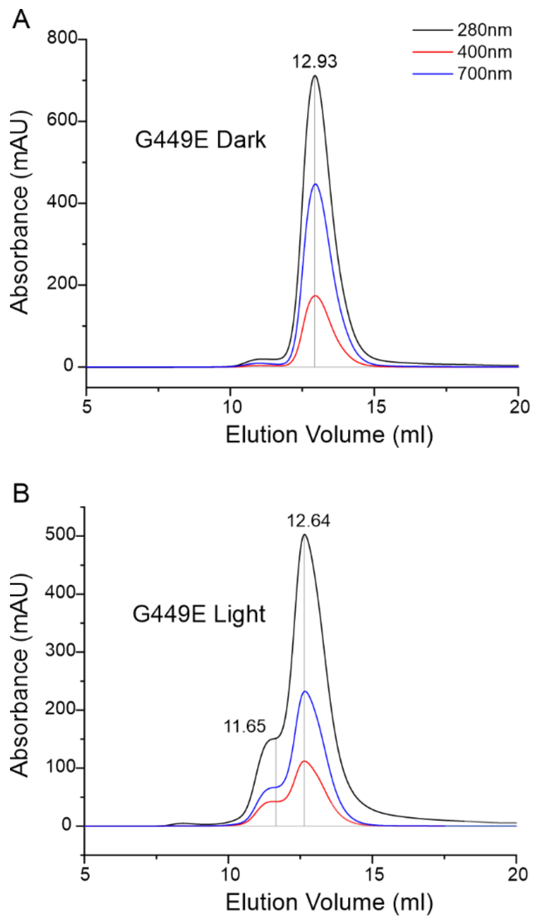

Supporting Figure S1. Gel-filtration analysis of the *Is*PadC G449E variant in the dark (A) and during constant red light illumination (B). In the dark, *Is*PadC G449E elutes as a dimer. Upon illumination, it features a mixture of dimer and higher oligomeric species. The black line corresponds to the absorbance at 280 nm, while the red and the blue lines represent wavelengths of 400 and 700 nm, respectively. The column used in this experiment is a Superdex 200 increase 10/300 GL (Cytiva).

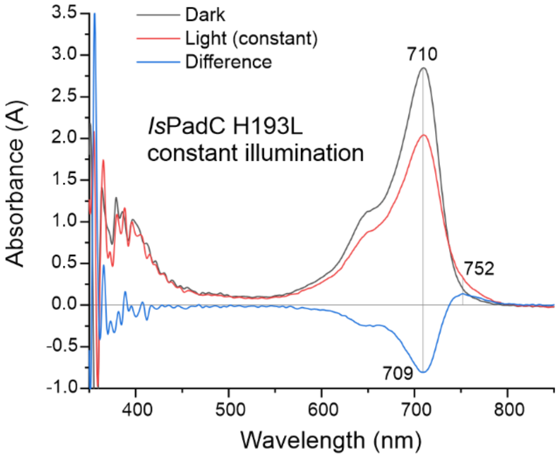

Supporting Figure S2. UV/vis absorption spectra of *Is*PadC H193L under constant red-light illumination measured on a Specord S300. The black and red lines represent the dark- and light-adapted states, respectively, and the difference spectrum (light *minus* dark) is in blue. The gray lines indicate the maxima and minima of the difference spectrum. The spectrum is scaled to the maximum Soret-band spectrum of the variant's dark state spectrum.

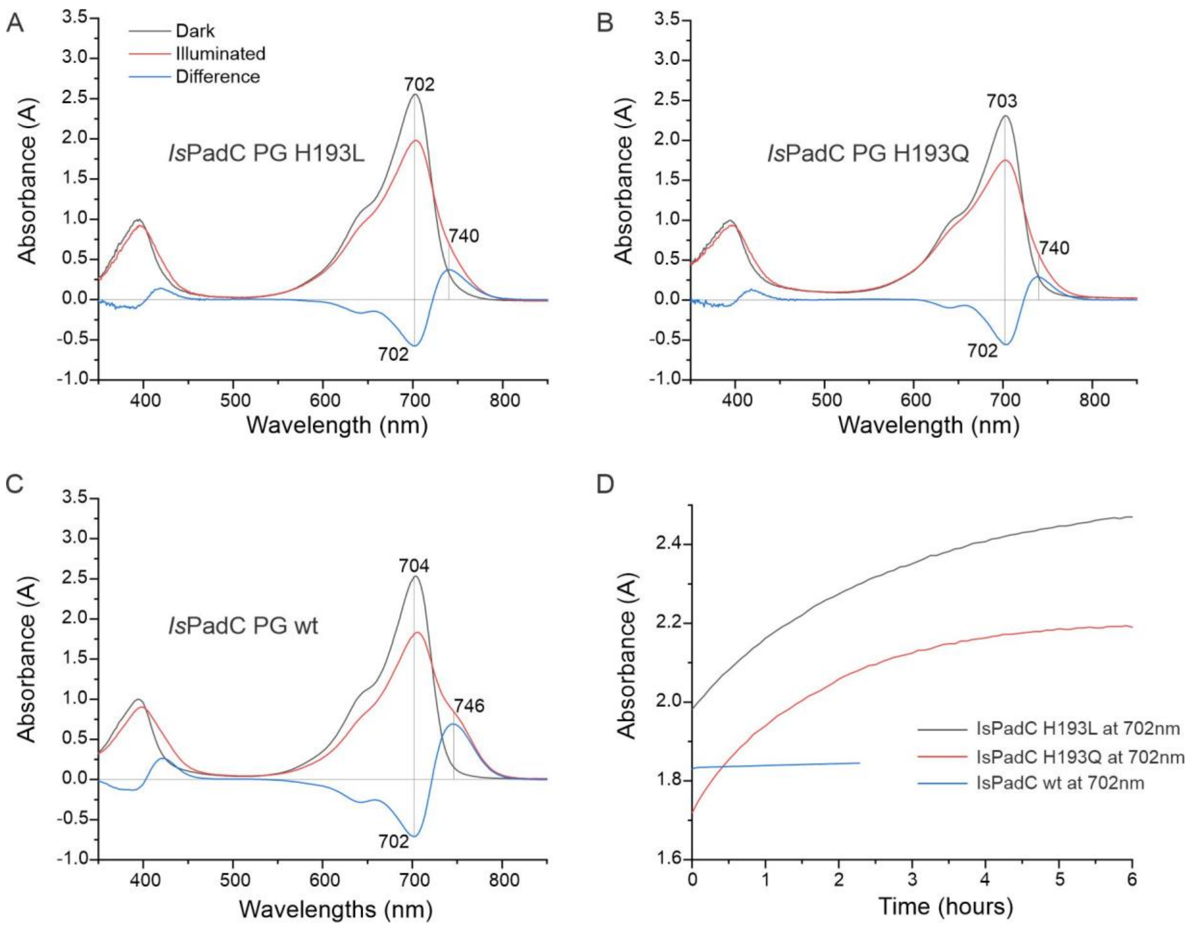

Supporting Figure S3. Spectral characterization of *Is*PadC PAS-GAF variants. **A - C**: UV/Vis absorption spectra of *Is*PadC PAS-GAF H193L, *Is*PadC PAS-GAF H193Q, and *Is*PadC PAS-GAF wt, respectively. The black line represents dark-adapted states, the red line represents light-adapted states, and the difference spectrum (light *minus* dark) is in blue. The gray lines indicate the maxima and minima of the difference spectra. Spectra are scaled based on the maximum Soret-band spectrum of each variant's dark state spectrum. **D**: Thermal recovery of *Is*PadC PASGAF H193L (black line), H193Q (red line), and wt (blue line).

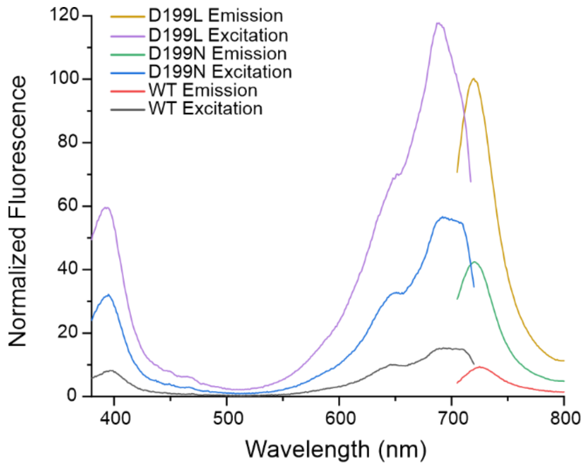

Supporting Figure S4. Fluorescence emission and excitation spectra of *Is*PadC variants compared to *Is*PadC wt. 2 µM samples were measured in triplicate with 740 nm and 685 nm for emission and excitation, respectively.

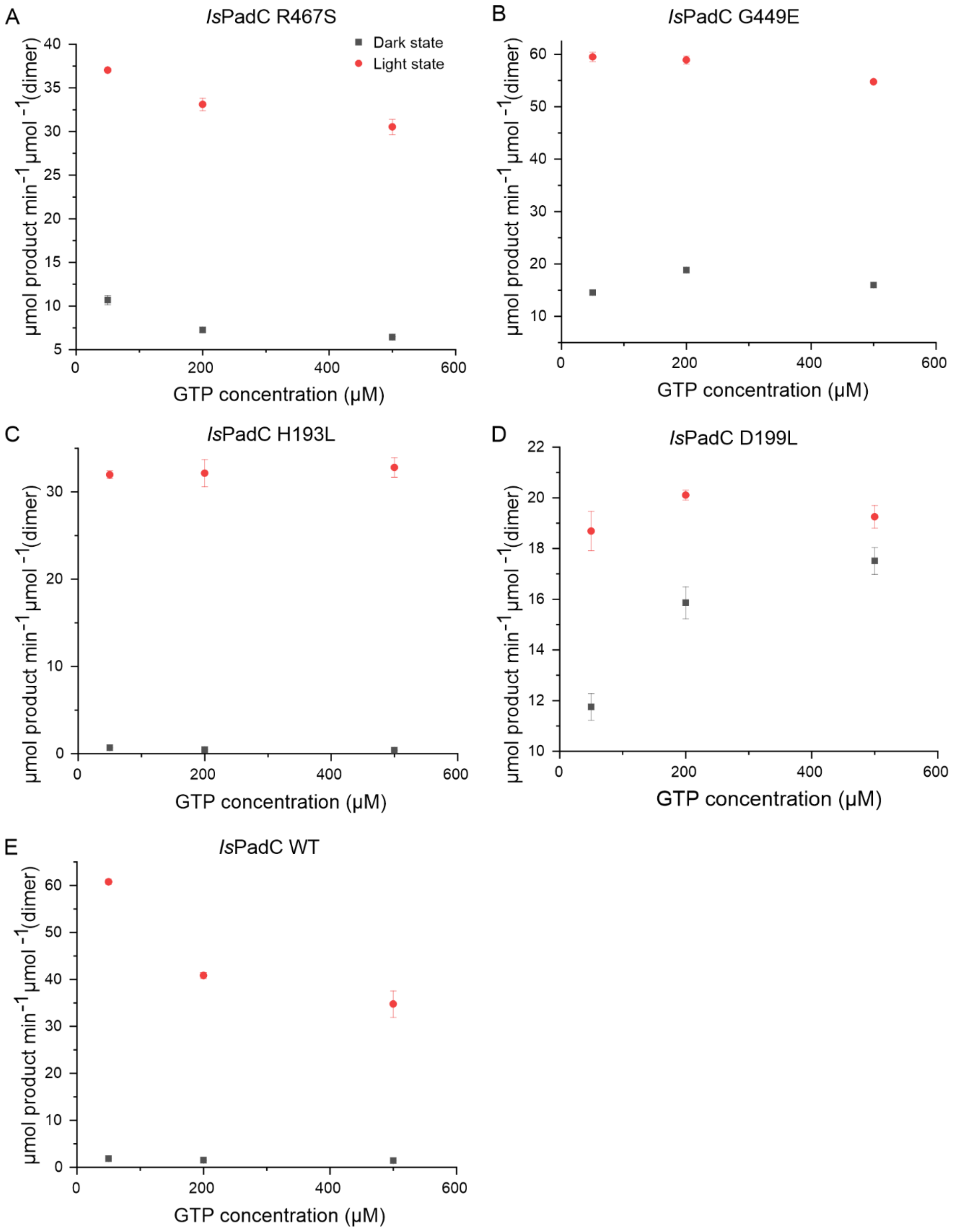

Supporting Figure S5. Kinetic characterization of GTP to c-di-GMP conversion of *Is*PadC variants (panel **A – D**) and *Is*PadC wt panel **E**. Initial velocities of product formation are plotted for different GTP starting concentrations. Apparent c-di-GMP turnover rates are normalized to the concentration of the dimeric proteins. Error bars show the error of the estimate for linear fits of c-di-GMP production weighted by the standard deviation from three experimental replicates.

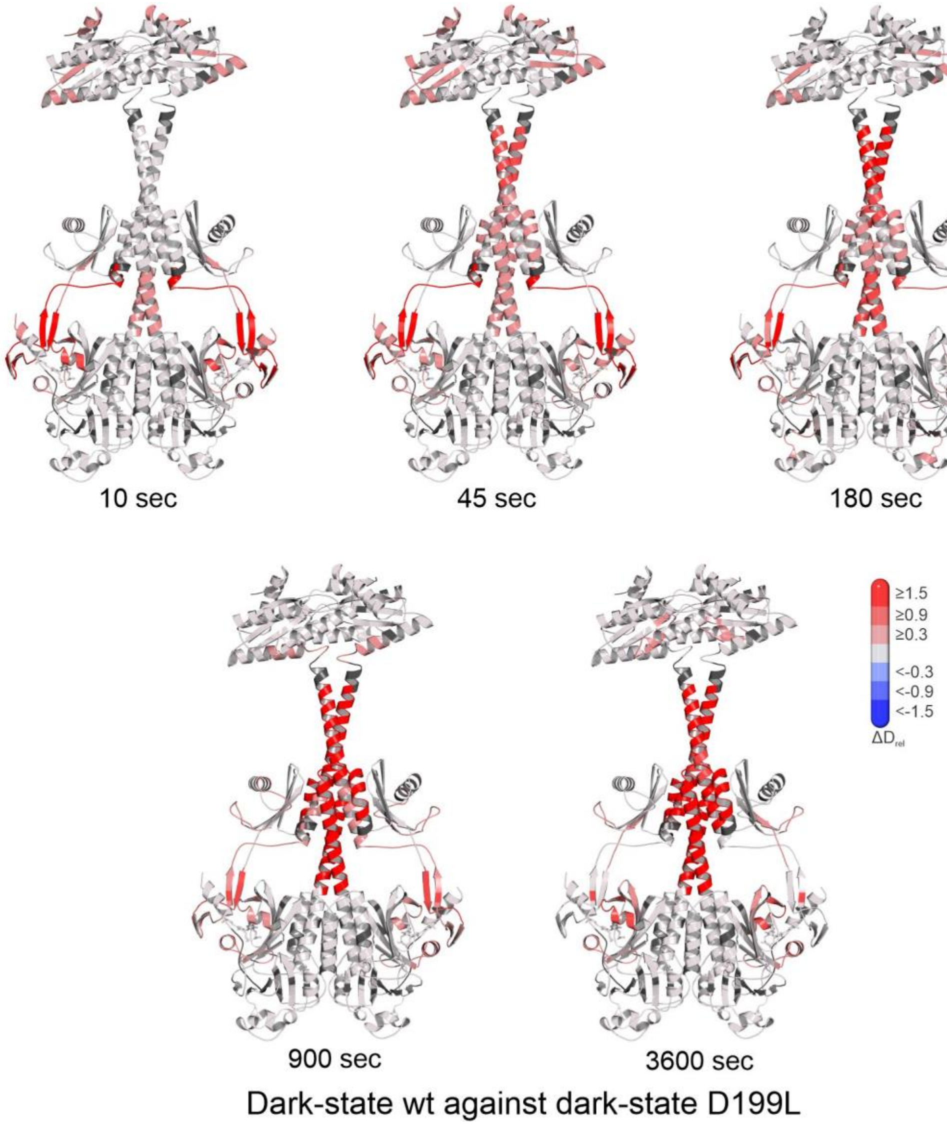

Supporting Figure S6. Comparison of the conformational dynamics between the dark-adapted state of *Is*PadC D199L and wild-type *Is*PadC as a reference. Differences in deuterium uptake at different time points are mapped onto the *Is*PadC wt structure (pdb: 5LLW). The coloring scheme corresponds to the scale bar at the lower right.

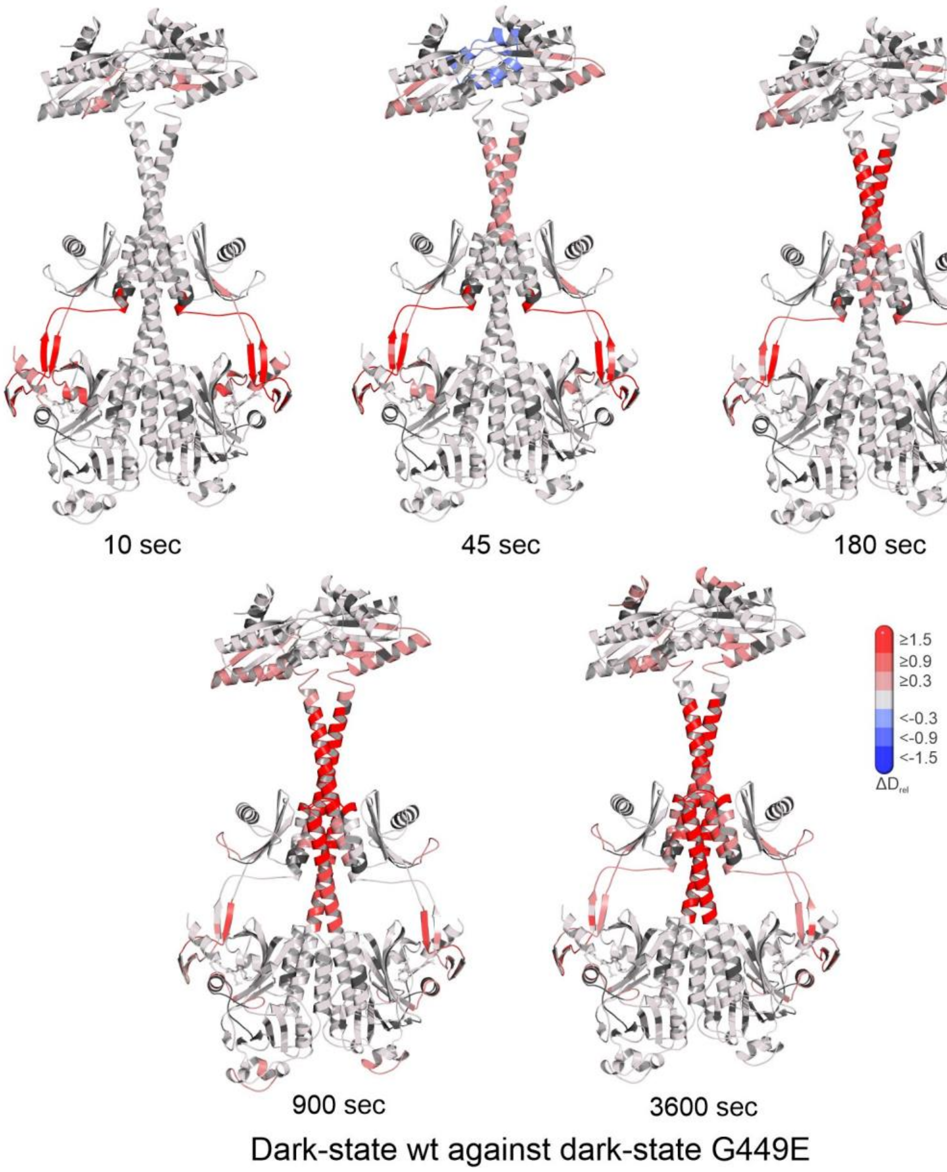

Supporting Figure S7. Comparison of the conformational dynamics between the dark-adapted state of *Is*PadC G449E and wild-type *Is*PadC as a reference. Differences in deuterium uptake at different time points are mapped onto the *Is*PadC wt structure (pdb: 5LLW). The coloring scheme corresponds to the scale bar at the lower right. The top right panel for the 180 s time point shows the same data as Figure 4A and is reused here for completeness.

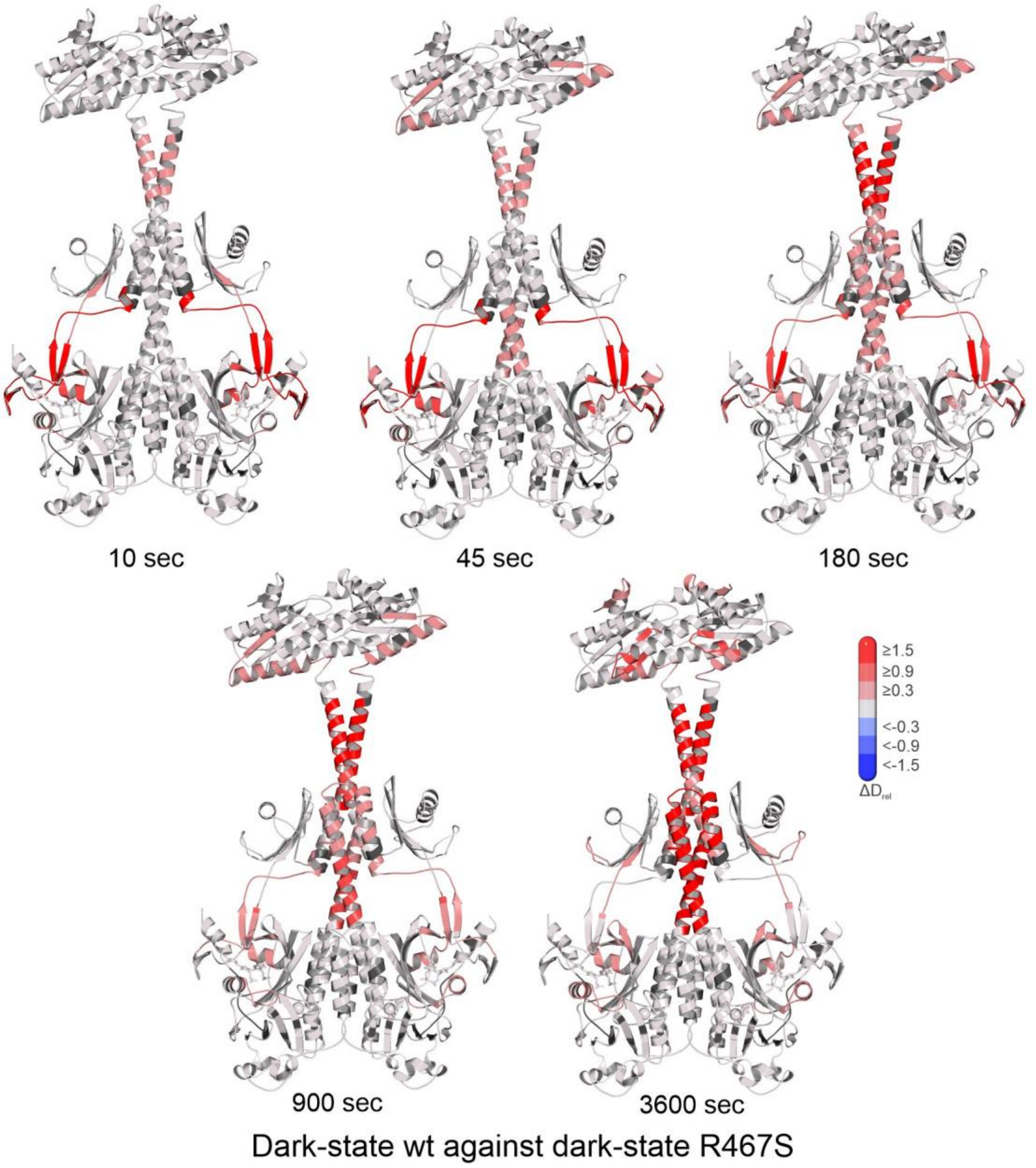

Supporting Figure S8. Comparison of the conformational dynamics between the dark-adapted state of *Is*PadC R467S and wild-type *Is*PadC as a reference. Differences in deuterium uptake at different time points are mapped onto the *Is*PadC wt structure (pdb: 5LLW). The coloring scheme corresponds to the scale bar at the lower right.

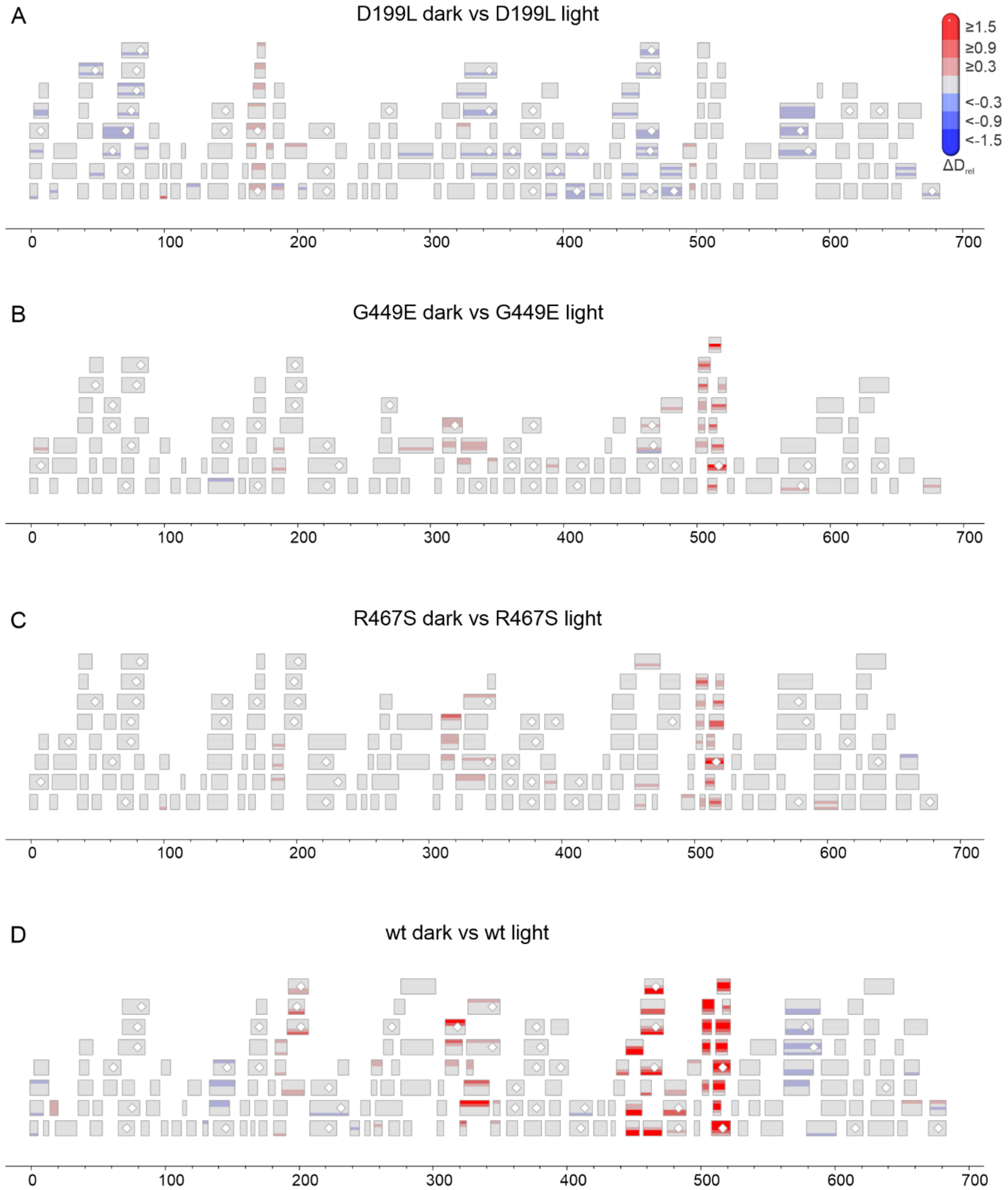

Supporting Figure S9. Overview of *Is*PadC peptides analyzed for different comparisons of HDX-MS data. Each box represents one peptide and consists of five horizontal bars that are colored according to the relative deuteration of two proteins compared. A: D199L light – D199L dark; B: G449E light – G449E dark; C: R467S light – R467S dark; D: wt light – wt dark. The bars correspond to the measured time points of 10 s, 3 min, 15 min, 45 min, and 60 min (bottom to top, respectively). The coloring scheme is according to the scale bar at the top right. Peptides marked with a diamond are MS^2 confirmed.

Supporting Table 1: Detail of all primers used for *Is*PadC variants

**Desired construct**	**Oligonucleotide (5´-3´)**	
*Is*PadC H139L	Fwd	GTTTACCAGCATGCTGAAT CTG CATTTTCCGGCAAGCGATATTCCG
	Rev	ATTCAGCATGCTGGTAAAC AGCTGACGAACGCTTTCTGCAATAACAC
*Is*PadC H139Q	Fwd	GTTTACCAGCATGCTGAAT CAG CATTTTCCGGCAAGCGATATTCCG
	Rev	ATTCAGCATGCTGGTAAAC AGCTGACGAACGCTTTCTGCAATAACAC
*Is*PadC D199L	Fwd	GCAAGCTTGATTCCGGCACAGGCACGTGCAATG
	Rev	CGGAATCAAGCTTGCCGGAAAATGATGATTCAGCATGCTG
*Is*PadC D199N	Fwd	CATCATTTTCCGGCAAGC AAT ATTCCGGCACAGGCACGTG
	Rev	GCTTGCCGGAAAATGATG ATTCAGCATGCTGGTAAACAGCTGACG
*Is*PadC G449E	Fwd	GTTCGTACCTGGGCA GAA AAACCGGAAAAACTGAGCGTTGAAACC
	Rev	TGCCCAGGTACGAAC TTCATTCTGTGCAACACGAAACAGCAGC
*Is*PadC R467S	Fwd	GGTCCGTCGAAAAGTTTTGAAGCATGGCAGGATGAAGTTAG
	Rev	ACTTTTCGACGGACCCAGCATGGTGCCGGTGC

Supporting Table 2: Details of all buffers used for purification, UV/vis and enzymatic characterization of *Is*PadC variants

**Buffer**	**Compositions**
Lysis buffer	50mM HEPES pH 7.0, 0.5M NaCl, 2mM $MgCl_2$, 10mM imidazole
Wash Buffer	50mM HEPES pH 7.0, 0.5M NaCl, 2mM $MgCl_2$, 50mM imidazole
Elution buffer	50mM HEPES pH 7.0, 0.5M NaCl, 2mM $MgCl_2$, 250mM imidazole
Dialysis buffer	50mM HEPES pH 7.0, 0.5M NaCl, 2mM $MgCl_2$
Gel-filtration buffer	10mM HEPES pH 7.0, 0.5M NaCl, 2mM $MgCl_2$
Reaction buffer	10mM HEPES pH 7.0, 0.5M NaCl, 50mM $MgCl_2$
Aqueous mobile phase	10 mM K_2HPO_4 pH 7.0, 1 mM EDTA, 6 % MeOH

Supporting Table 3. Details of all acquired HDX datasets according to conventions of the HDX-MS community.

	***Is*PadC wild-type**		**D199L**		**G449E**		**R467S**	
Light conditions	Dark	Light	Dark	Light	Dark	Light	Dark	Light
HDX reaction details	10 mM HEPES, 150 mM NaCl, 2 mM $MgCl_2$, pD = 7.0, 20 °C							
HDX time course (s)	10, 45, 180, 900, 3600							
HDX control samples	Unlabeled control (wt dark)		Unlabeled control (D199L dark)		Unlabeled control (G449E dark)		Unlabeled control (R467S dark)	
Back-exchange	not measured							
# of Peptides	203	229	235	192	144	156	220	190
Sequence coverage	96%	97%	96%	94%	90%	92%	95%	94%
Avg. peptide length/ Average Redundancy	12 / 3.5	12 / 4.1	13 / 4.4	11 / 3.2	12 / 2.4	12 / 2.7	12 / 3.8	12 / 3.2
Replicates	3	3	3	3	3	3	3	3
Repeatability (average SD for each time point)	0.05, 0.04, 0.04, 0.06, 0.10	0.05, 0.11, 0.16, 0.17, 0.16	0.05, 0.07, 0.06, 0.09, 0.09	0.07, 0.09, 0.14, 0.19, 0.17	0.05, 0.05, 0.09, 0.09, 0.13	0.04, 0.05, 0.04, 0.05, 0.06	0.07, 0.07, 0.05, 0.08, 0.09	0.05, 0.05, 0.06, 0.12, 0.17
Sign. Diff. in HDX	∆HDX > 0.3 D							
